# The prognostic value of SUMO1/Sentrin specific peptidase 1 (SENP1) in prostate cancer is limited to ERG-fusion positive tumors lacking PTEN deletion

**DOI:** 10.1186/s12885-015-1555-8

**Published:** 2015-07-23

**Authors:** Christoph Burdelski, Devi Menan, Maria Christina Tsourlakis, Martina Kluth, Claudia Hube-Magg, Nathaniel Melling, Sarah Minner, Christina Koop, Markus Graefen, Hans Heinzer, Corinna Wittmer, Guido Sauter, Ronald Simon, Thorsten Schlomm, Stefan Steurer, Till Krech

**Affiliations:** 1 General, Visceral and Thoracic Surgery Department and Clinic, University Medical Center Hamburg-Eppendorf, Hamburg, Germany; 2 Institute of Pathology, University Medical Center Hamburg-Eppendorf, Hamburg, Germany; 3 Martini-Clinic, Prostate Cancer Center, University Medical Center Hamburg- Eppendorf, Martinistr. 25, 20246 Hamburg, Germany; 4 Department of Urology, Section for translational Prostate Cancer Research, University Medical Center Hamburg-Eppendorf, Hamburg, Germany

**Keywords:** Prostate cancer, ERG fusion, *PTEN* deletion, SENP1, SUMO, Immunohistochemistry, Tissue microarray

## Abstract

**Background:**

Posttranscriptional protein modification by SUMOylation plays an important role in tumor development and progression. In the current study we analyzed prevalence and prognostic impact of the de-SUMOylation enzyme SENP1 in prostate cancer.

**Methods:**

SENP1 expression was analyzed by immunohistochemistry on a tissue microarray containing more than 12,400 prostate cancer specimens. Results were compared to tumor phenotype, ERG status, genomic deletions of 3p, 5q, 6q and *PTEN*, and biochemical recurrence.

**Results:**

SENP1 immunostaining was detectable in 34.5 % of 9,516 interpretable cancers and considered strong in 7.3 %, moderate in 14.9 % and weak in 12.3 % of cases. Strong SENP1 expression was linked to advanced pT stage (p < 0.0001), high Gleason grade (p < 0.0001), positive lymph node status (*p =* 0.0019), high pre-operative PSA levels (*p =* 0.0037), and PSA recurrence (p < 0.0001). SENP1 expression was strongly associated with positive ERG fusion status as determined by both in situ hybridization (FISH) and immunohistochemistry as well as with *PTEN* deletions. Detectable SENP1 immunostaining was found in 41 % of ERG positive and in 47 % of *PTEN* deleted cancers but in only 30 % of ERG negative and 30 % of *PTEN* non-deleted cancers (p < 0.0001 each). Deletions of 3p, 5q, and 6q were unrelated to SENP1 expression. Subset analyses revealed that the prognostic impact of SENP1 expression was solely driven by the subgroup of ERG positive, *PTEN* undeleted cancers. In this subgroup, the prognostic role of SENP1 expression was independent of the preoperative PSA level, tumor stage, Gleason grade, and the status of the resection margin.

**Conclusions:**

SENP1 expression has strong prognostic impact in a molecularly defined subset of cancers. This is per se not surprising as the biologic impact of each individual molecular event is likely to be dependent on its cellular environment. However, such findings challenge the concept of finding clinically relevant molecular signatures that are equally applicable to all prostate cancers.

**Electronic supplementary material:**

The online version of this article (doi:10.1186/s12885-015-1555-8) contains supplementary material, which is available to authorized users.

## Background

Prostate cancer is the most prevalent cancer in men in Western societies [1]. Although most prostate cancers have a rather indolent clinical course, this disease still represents the third most common cause of cancer related death in men. A reliable distinction between the indolent and the aggressive forms of the disease is highly desirable to enhance therapeutic decisions. Despite recent advances, the only established pretreatment prognostic parameters currently include Gleason grade and tumor extent on biopsies, preoperative prostate-specific antigen (PSA), and clinical stage. Because these data are statistically powerful but not sufficient for optimal individual treatment decisions, it can be hoped that a better understanding of disease biology will eventually lead to the identification of clinically applicable molecular markers that enable a more reliable prediction of prostate cancer aggressiveness.

SUMOylation is a revertible posttranscriptional protein modification involving the binding of small ubiquitin-like modifiers (SUMOs) to target proteins. SUMOs are structurally related to ubiquitin and are covalently attached to target proteins by a SUMO-conjugating system resembling the ubiquitination machinery [2]. SUMOylation affects protein stability and activity, and regulates a variety of cellular processes, such as nuclear transport, transcription, and apoptosis [3]. Several proteins control the balance between SUMOylation and de-SUMOylation. A key protein for de-SUMOylation is SUMO1/Sentrin specific peptidase 1 (SENP1) [4], which deconjugates SUMOs from a large number of SUMOylated proteins [5]. Since important target genes of SENP1 include histone deacetylases and cell cycle regulators like cyclin D1, SENP1 is also involved in control of epigenetic transcription and cell proliferation [6–10]. Consequently, overexpression of SENP1 has been found in various cancer types [10], such as colon cancer [11], bladder cancer [12], head & neck cancer [13], and lung cancer [14], and has been linked to poor clinical features in some of these [13, 15]. In the prostate gland, SENP1 was shown to act as a transcriptional activator of androgen receptor (AR) signaling [7]. Two studies analyzing SENP1 in 115 and 150 Asian prostate cancer patients suggested that SENP1 overexpression might be an independent marker of poor prognosis [16, 17].

These promising findings prompted us to study the putative prognostic value of SENP1 expression measurement in a large cohort including more than 12,400 European prostate cancers that have been assembled in a tissue microarray (TMA) format. The database attached to our TMA contains pathological and clinical follow-up data, as well molecular data of key molecular alterations of this disease such as ERG fusion and genomic deletion of *PTEN*, 3p13, 5q21, and 6q15, which were used to establish associations between SENP1 expression and distinct phenotypic and molecular subsets of prostate cancers.

## Methods

### Patients

Radical prostatectomy specimens were available from 12,427 patients, undergoing surgery between 1992 and 2012 at the Department of Urology and the Martini Clinics at the University Medical Center Hamburg-Eppendorf. Follow-up data were available for a total of 11,665 patients with a median follow-up of 36 months (range: 1 to 241 months; Table 1). Prostate specific antigen (PSA) values were measured following surgery and PSA recurrence was defined as a postoperative PSA of ≥ 0.2 ng/ml confirmed by a second determination with a serum PSA ≥ 0.2 ng/ml. All prostate specimens were analyzed according to a standard procedure, including a complete embedding of the entire prostate for histological analysis [18].

**Table 1:** Pathological and clinical data of the arrayed prostate cancers. Percentage in the column “Study cohort on TMA” refers to the fraction of samples across each category. Percentage in column “Biochemical relapse among categories” refers to the fraction of samples with biochemical relapse within each parameter in the different categories. Numbers do not always add up to 12,427 in the different categories because of cases with missing data. Abbreviation: AJCC, American Joint Committee on Cancer

	No. of patients (%)	
	Study cohort on TMA (*n =* 12427)	Biochemical relapse among categories
Follow-up (mo)		
n	11665 (93.9 %)	2769 (23.7 %)
Mean	48.9	-
Median	36.4	-
Age (y)		
≤50	334 (2.7 %)	81 (24.3 %)
51-59	3061 (24.8 %)	705 (23 %)
60-69	7188 (58.2 %)	1610 (22.4 %)
≥70	1761 (14.3 %)	370 (21 %)
Pretreatment PSA (ng/ml)		
<4	1585 (12.9 %)	242 (15.3 %)
4-10	7480 (60.9 %)	1355 (18.1 %)
10-20	2412 (19.6 %)	737 (30.6 %)
>20	812 (6.6 %)	397 (48.9 %)
pT category (AJCC 2002)		
pT2	8187 (66.2 %)	1095 (13.4 %)
pT3a	2660 (21.5 %)	817 (30.7 %)
pT3b	1465 (11.8 %)	796 (54.3 %)
pT4	63 (0.5 %)	51 (81 %)
Gleason grade		
≤3 + 3	2983 (24.1 %)	368 (12.3 %)
3 + 4	6945 (56.2 %)	1289 (18.6 %)
4 + 3	1848 (15 %)	788 (42.6 %)
≥4 + 4	584 (4.7 %)	311 (53.3 %)
pN category		
pN0	6970 (91 %)	1636 (23.5 %)
pN+	693 (9 %)	393 (56.7 %)
Surgical margin		
Negative	9990 (81.9 %)	1848 (18.5 %)
Positive	2211 (18.1 %)	853 (38.6 %)

The TMA manufacturing process was described earlier in detail [19]. In short, one 0.6 mm core was taken from a representative tissue block from each patient. The tissues were distributed among 27 TMA blocks, each containing 144 to 522 tumor samples. For internal controls, each TMA block also contained various control tissues, including normal prostate tissue. The molecular database attached to this TMA contained results on ERG expression in 10,711 [20], *ERG* break apart FISH analysis in 7,122 (expanded from [21]) and deletion status of 5q21 (*CHD1*) in 7932 (expanded from [22]), 6q15 (*MAP3K7*) in 6,069 (expanded from [23]), 10q23 (*PTEN*) in 6,704 (expanded from [24]) and 3p13 (*FOXP1*) in 7,081 (expanded from [25]) cancers. Immunohistochemical data on Ki67 labeling index (LI) were available from 7,010 cancers (expanded from [26]).

The usage of archived diagnostic left-over tissues for manufacturing of tissue microarrays and their analysis for research purposes as well as patient data analysis has been approved by the local ethics committee (Ethics commission Hamburg, WF-049/09 and PV3652). All work has been carried out in compliance with the Helsinki Declaration.

Usage of patient data and routinely archived formalin fixed left-over patient tissue samples for research purposes by the attending physician is approved by local laws and does not require written consent (HmbKHG, §12,1).

### Immunohistochemistry

Freshly cut TMA sections were immunostained on one day and in one experiment. Slides were deparaffinized and exposed to heat-induced antigen retrieval for 5 min in an autoclave at 121 °C in pH 7.8 Tris-EDTA-Citrate buffer. Primary antibody specific for SENP1 (rabbit monoclonal antibody, EPR3844, Abcam, Cambridge, UK; cat#108981; dilution 1:150) was applied at 37 °C for 60 min. Bound antibody was then visualized using the EnVision Kit (Dako, Glostrup, Denmark) according to the manufacturer’s directions. Staining was predominantly nuclear and typically accompanied by cytoplasmic co-staining. The intensity of the cytoplasmic staining was usually weaker than the intensity of nuclear staining. Nuclear and cytoplasmic SENP1 staining was typically found in either all (100 %) or none (0 %) of the tumor cells in a given cancer spot. Staining intensity of all cases was thus semi-quantitatively assessed in four categories: negative, weak, moderate and strong. The percentage of positive tumor cells (typically 100 %) was not separately recorded. An additional isotype control (rabbit monoclonal, SP137, Abcam, Cambridge, UK; cat#128142) yielded no unspecific staining (data not shown).

### Statistics

For statistical analysis, the JMP® 10.0.2 software (2012 SAS Institute Inc., NC, USA) was used. Contingency tables were calculated to study association between SENP1 staining and clinico-pathological variables, and the Chi-squared (Likelihood) test was used to find significant relationships. Kaplan Meier curves were generated for PSA recurrence free survival. The log-Rank test was applied to test the significance of differences between stratified survival functions. Cox proportional hazards regression analysis was performed to test the statistical independence and significance between pathological, molecular, and clinical variables.

## Results

### Technical issues

A total of 9,516 (77 %) of tumor samples were interpretable in our TMA analysis. Reason for non-informative cases (2,911 spots; 23 %) included lack of tissue samples or absence of unequivocal cancer tissue in the TMA spot.

### SENP1 immunohistochemistry

**Fig. 1:**
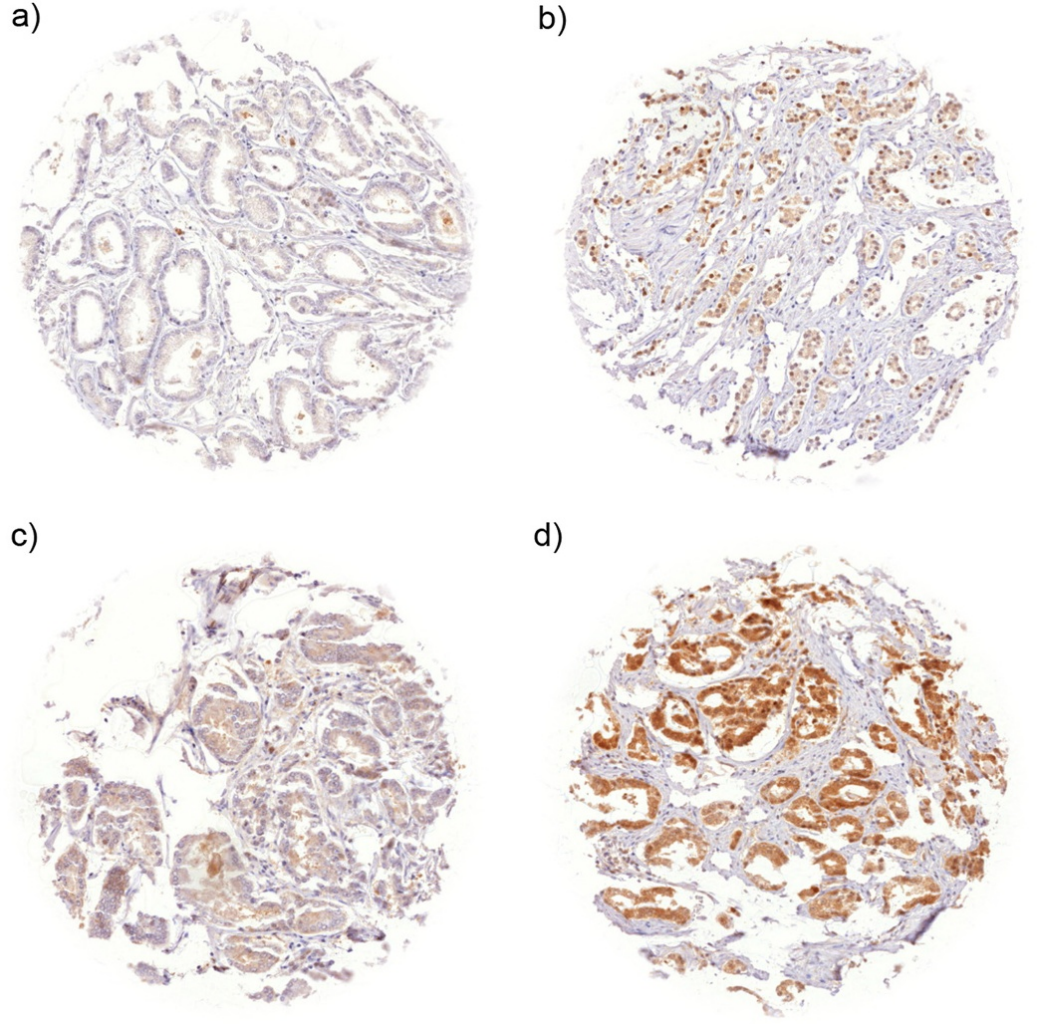
Representative pictures of SENP1 immunostaining in prostate cancer with **a**) negative, **b**) weak, **c**) moderate, and **d**) strong staining

In normal prostatic glands, weak cytoplasmic staining was found in almost all cases, whereas nuclear staining was rare and occurred in only two out of 20 (10 %) cases. Positive staining was limited to the secretory epithelial cells, while basal cells were consistently negative. In cancers, SENP1 immunostaining was predominantly localized in the nucleus. Positive staining was seen in 3,283 of our 9,516 (34.5 %) interpretable tumors and was considered weak in 12.3 %, moderate in 14.9 % and strong in 7.3 % of cancers. Representative images of positive and negative SENP1 immunostainings are given in Fig. 1. Strong SENP1 immunostaining was significantly linked to advanced pathological tumor stage (p < 0.0001), high Gleason grade (p < 0.0001), presence of lymph node metastases (*p =* 0.0019) and high preoperative PSA-levels (*p =* 0.0037) when all tumors were jointly analyzed (Table 2). SENP1 immunostaining showed no significant association with positive resection margin status (*p =* 0.3216).

**Table 2:** Association between SENP1 immunostaining results and prostate cancer phenotype in all cancers

Parameter	SENP1					p value
	n evaluable	Negative (%)	Weak (%)	Moderate (%)	Strong (%)	
All cancers	9,516	65.5	12.3	14.9	7.3	
Tumor stage						*<0.0001*
pT2	6,143	68.2	11.1	14.1	6.6	
pT3a	2,137	61.8	13.7	15.9	8.7	
pT3b-4	1,203	58.1	15.8	17.6	8.5	
Gleason grade						*<0.0001*
≤3 + 3	2,135	72.5	9.2	11.6	6.7	
3 + 4	5,451	65.3	11.7	15.5	7.5	
4 + 3	1,445	58.4	16.2	17.6	7.8	
≥4 + 4	442	58.1	20.8	14.9	6.1	
Lymph node metastasis						*0.0019*
N0	5,472	62.3	12.6	16.6	8.5	
N+	526	56.8	18.4	17.5	7.2	
Preop. PSA level (ng/ml)						*0.0037*
<4	1160	64.2	12.2	15.9	7.6	
4-10	5702	66.8	11.2	15.0	7.0	
10-20	1892	63.4	14.6	14.5	7.5	
>20	666	62.3	14.9	14.3	8.6	
Surgical margin						*0.3216*
negative	7,549	65.9	12.1	14.9	7.1	
positive	1,797	63.8	13.0	15.2	8.0	

### Association with *TMPRSS2:ERG* fusion status and ERG protein expression

**Fig. 2:**
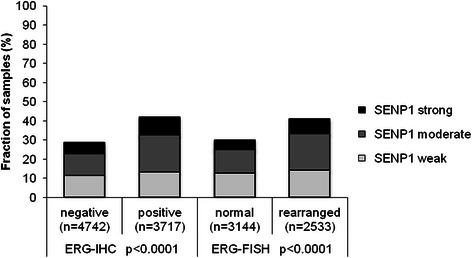
Association between SENP1 immunostaining results and the ERG-status determined by IHC and FISH analysis. Rearranged indicates breakage of the ERG gene according to FISH analysis

To evaluate whether SENP1 expression is associated with ERG status in prostate cancers, we used data from previous studies (expanded from [20, 21]). Data on *TMPRSS2:ERG* fusion status obtained by FISH were available from 5,677 and by immunohistochemistry from 8,459 tumors with evaluable SENP1 immunostaining. Data on both ERG FISH and IHC were available from 5,468 cancers, and an identical result (ERG IHC positive and break by FISH or ERG IHC negative and missing break by FISH) was found in 5,231 of 5,468 (95.7 %) cancers. SENP1 immunostaining was slightly more frequent in *TMPRSS2:ERG* rearranged and ERG positive prostate cancers than in ERG negative tumors. Positive SENP1 immunostaining was seen in 41.7 % (ERG IHC) and 40.9 % (*ERG* FISH) of ERG positive cancers but in only 28.6 % and 30 % of cancers without ERG staining and *ERG* rearrangement, respectively (p < 0.0001 each; Fig. 2). SENP1 immunostaining was similarly linked to unfavorable tumor features in subsets of both ERG negative and ERG positive cancers (Additional file 1: Table S1 and Additional file 2: Table S2).

### Association to other key genomic deletions

**Fig. 3:**
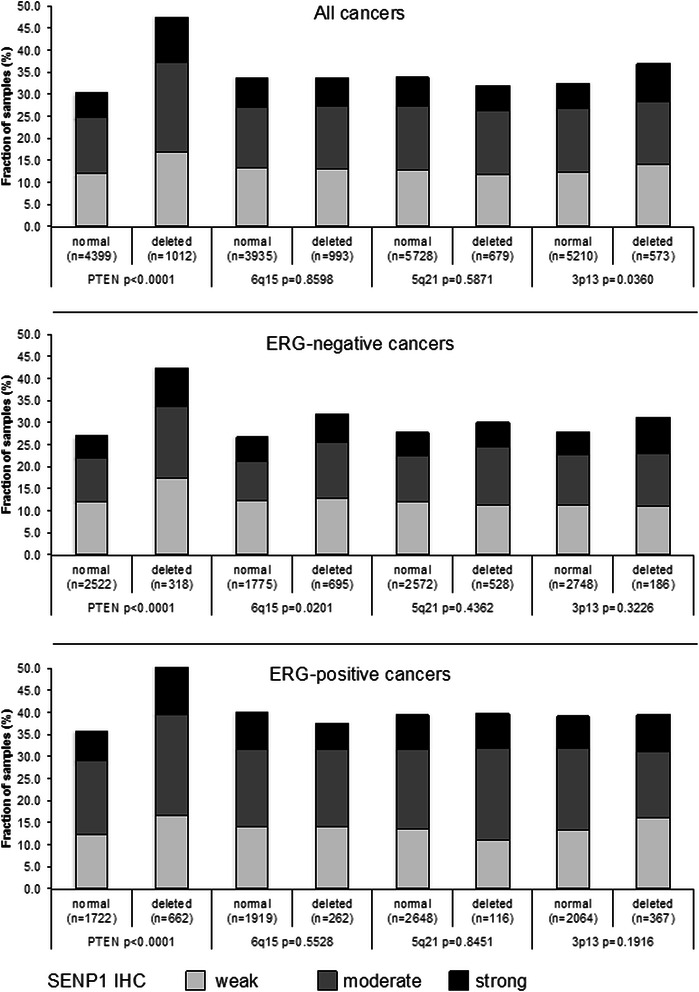
Association between positive SENP1 immunostaining results and deletions of *PTEN*, 5q21 (*CHD1*), 6q15 (*MAP3K7*), and 3p13 (*FOXP1*) in all cancers as well as the subsets of ERG-negative and ERG-positive cancers according to ERG-IHC analysis

Earlier studies had provided evidence for recurrent chromosomal deletions delineating further molecular subgroups amongst ERG positive and ERG negative prostate cancers. In particular, deletions of *PTEN* and 3p13 define subgroups in ERG positive and deletions of 5q21 and 6q15 define subgroups in ERG negative cancers [22, 23, 25]. To examine, whether SENP1 expression might be particularly associated with one of these genomic deletions, SENP1 data were compared to preexisting findings on *PTEN* (10q23), 3p13 (*FOXP1*), 6q15 (*MAP3K7*) and 5q21 (*CHD1*) deletions. Elevated SENP1 expression levels were strongly linked to deletions of *PTEN* both in ERG positive and ERG negative cancers (p < 0.0001 each, Fig. 3). However, SENP1 was largely unrelated to all other deletions irrespective of whether all cancers or subgroups of ERG positive or ERG negative cancers were analyzed.

### Association to tumor cell proliferation (Ki67LI)

Strong SENP1 staining was significantly linked to accelerated cell proliferation as measured by Ki67LI in all cancers (p < 0.0001). This association held also true with high significance in most subgroups of cancers with identical Gleason grade (≤3 + 3; 3 + 4; 4 + 3; ≥4 + 4), and also in the subset of ERG positive tumors lacking *PTEN* deletions (*p =* 0.0315). All comparisons with the Ki67LI are summarized in Table 3.

**Table 3:** Associations between SENP1 immunohistochemistry results and cell proliferation as measured by Ki67 immunohistochemistry in all cancers and subsets of cancers defined by Gleason grade, and the ERG/PTEN status. Ki67LIav = average Ki67 labeling index. * *P*-value for SENP1 negative vs. positive (combined groups of weak, moderate, strong)

		SENP1 IHC	Number	Ki67LI av		*P*
All cancers		negative	3,880	2.58	±0.04	
		weak	679	3.05	±0.10	<0.0001
		moderate	838	3.31	±0.09	* < 0.0001
		strong	419	3.21	±0.13	
Gleason	≤3 + 3	negative	980	2.07	±0.07	
		weak	112	2.30	±0.19	<0.0001
		moderate	137	2.55	±0.18	* < 0.0001
		strong	75	2.65	±0.24	
	3 + 4	negative	2,238	2.51	±0.05	
		weak	396	3.02	±0.12	<0.0001
		moderate	520	3.14	±0.10	* < 0.0001
		strong	252	3.24	±0.15	
	4 + 3	negative	504	3.34	±0.16	
		weak	119	3.69	±0.32	0.4329
		moderate	137	3.85	±0.30	*0.1209
		strong	71	3.56	±0.42	
	≥4 + 4	negative	133	4.74	±0.39	
		weak	51	3.41	±0.63	0.0516
		moderate	41	5.90	±0.70	*0.5643
		strong	18	3.78	±1.06	
ERG-positive cancers without PTEN deletion		negative	814	2.92	±0.09	
		weak	151	3.44	±0.21	0.0315
		moderate	196	2.99	±0.19	*0.0293
		strong	80	3.59	±0.29	

### Association with PSA recurrence

**Fig. 4:**
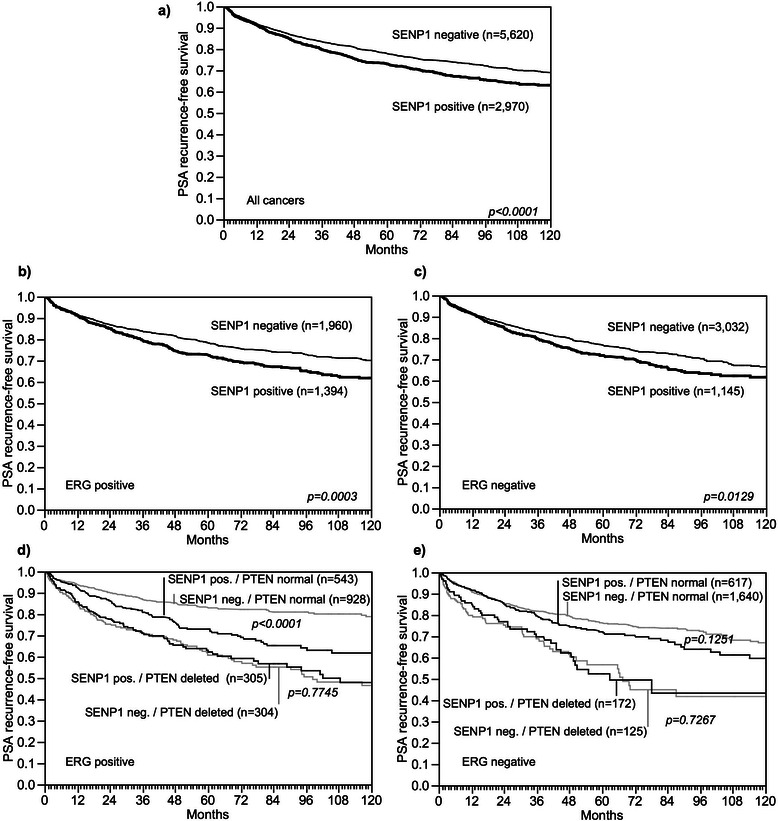
Association between SENP1 expression and biochemical recurrence in **a**) all cancers, **b**) ERG-IHC positive cancers, **c**) ERG-IHC negative cancers. Combined effect of SENP1 and PTEN deletion in **d**) all cancers, **e**) ERG-IHC positive cancers and **f**) ERG-IHC negative cancers

Follow-up data were available from 8,920 patients with interpretable SENP1 immunostaining on the TMA. Since there was no significant prognostic impact of the level of positive SENP1 staining (data not shown), all cancers with weak, moderate, and strong SENP1 staining were combined into one group (“positive”) for follow-up analysis. Tumors with positive SENP1 immunostaining showed a significantly shortened PSA recurrence-free interval if all cancers were jointly analyzed (p < 0.0001, Fig. 4a), as well as in subsets of ERG-IHC-positive (p < 0.0001, Fig. 4b) or ERG-IHC-negative cancers p < 0.0001, Fig. 4c). Because of the strong link between SENP1 expression and *PTEN* deletion, we extended the analyses to tumor subgroups stratified according to the SENP1/ *PTEN* status. These analyses revealed that the prognostic impact of SENP1 expression was limited to cancers lacking *PTEN* deletions in ERG positive (p < 0.0001 Fig. 4d), but not in ERG negative tumors (*p =* 0.1251, Fig. 4e). SENP1 had no prognostic relevance in cancers harboring *PTEN* deletions, neither in ERG positive (*p =* 0.7745, Fig. 4d), nor in ERG negative cancers (*p =* 0.7267, Fig. 4e).

### Multivariate analysis

Four different types of multivariate analyses were performed evaluating the clinical relevance of SENP1 expression in different scenarios (Table 4). Scenario 1 evaluated all postoperatively available parameters including pathological tumor stage, pathological lymph node status (pN), surgical margin status, preoperative PSA value and pathological Gleason grade obtained after the morphological evaluation of the entire resected prostate. In scenario 2, all postoperatively available parameters with exception of nodal status were included. The rational for this approach was that the indication and extent of lymph node dissection is not standardized in the surgical therapy of prostate cancer and that excluding pN in multivariate analysis can markedly increase case numbers. Two additional scenarios had the purpose to model the preoperative situation as much as possible. Scenario 3 included SENP1 expression, preoperative PSA, clinical tumor stage (cT stage) and Gleason grade obtained on the prostatectomy specimen. Since postoperative determination of a tumors Gleason grade is “better” than the preoperatively determined Gleason grade (subjected to sampling errors and consequently under-grading in more than one third of cases [27]), another multivariate analysis was added. In scenario 4, the preoperative Gleason grade obtained on the original biopsy was combined with preoperative PSA, cT stage and SENP1 expression. SENP1 largely did not provide independent prognostic information if all tumors or the subgroups of ERG positive and ERG negative cancers were interrogated. A further subset analysis of ERG positive/*PTEN* undeleted cancers revealed independent prognostic impact, however, in 3 of 4 tested scenarios (Table 4 a-d).

**Table 4:** Multivariate analysis including SENP1 expression in a) all cancers, b) ERG-negative, c) ERG-positive cancers and d) ERG-positive cancers lacking PTEN deletion

a)									
Scenario	n analyzable	p -value							
		Preoperative PSA-Level	pT Stage	cT Stage	Gleason grade prostatectomy	Gleason grade biopsy	N-Stage	R-Status	SENP1 Expression
1	5,273	<0.0001	<0.0001	-	<0.0001	-	0.0001	0.0008	0.9255
2	8,392	<0.0001	<0.0001	-	<0.0001	-	-	<0.0001	0.9136
3	8,268	<0.0001	-	<0.0001	<0.0001	-	-	-	0.6842
4	8,155	<0.0001	-	<0.0001	-	<0.0001	-	-	0.0227
b)									
Scenario	n analyzable	p -value							
		Preoperative PSA-Level	pT Stage	cT Stage	Gleason-grade prostatectomy	Gleason grade biopsy	N-Stage	R-Status	SENP1 Expression
1	2,681	0.0004	<0.0001	-	<0.0001	-	0.0006	0.1375	0.1487
2	4,179	<0.0001	<0.0001	-	<0.0001	-	-	0.0022	0.1962
3	4,145	<0.0001	-	<0.0001	<0.0001	-	-	-	0.3180
4	4,091	<0.0001	-	<0.0001	-	<0.0001	-	-	0.3539
c)									
Scenario	n analyzable	p -value							
		preoperative PSA-Level	pT Stage	cT Stage	Gleason-grade prostatectomy	Gleason grade biopsy	N-Stage	R-Status	SENP1 Expression
1	2,090	0.0003	<0.0001	-	<0.0001	-	0.0073	0.0065	0.4108
2	3,279	<0.0001	<0.0001	-	<0.0001	-	-	<0.0001	0.4351
3	3,203	<0.0001	-	<0.0001	<0.0001	-	-	-	0.3231
4	3,156	<0.0001	-	<0.0001	-	<0.0001	-	-	0.0143
d)									
Scenario	n analyzable	p -value							
		preoperative PSA-Level	pT Stage	cT Stage	Gleason-grade prostatectomy	Gleason grade biopsy	N-Stage	R-Status	SENP1 Expression
1	872	0.0015	<0.0001	-	<0.0001	-	0.0007	0.0958	0.1174
2	1,495	0.0012	<0.0001	-	<0.0001	-	-	0.0017	0.0157
3	1,463	<0.0001	-	0.0197	<0.0001	-	-	-	0.0057
4	1,444	<0.0001	-	0.0354	-	<0.0001	-	-	0.0005

## Discussion

Immunohistochemically detectable SENP1 expression was found in about 35 % of prostate cancers in our study. This frequency is lower than what has been observed in two earlier IHC studies, reporting positive SENP1 staining in 76.5 % of 115 [16] and high SENP1 expression in 47 % of 117 [17] analyzed prostate cancers from Asian patients. These earlier studies also analyzed tissue microarrays. Although both previous studies utilized a slightly larger core diameter (1 mm) than in our study (0.6 mm), it seems unlikely that the lower fraction of SENP1 positive cancers in our study was caused by sampling bias due to this small difference in core diameter. Rather, different antibodies, immunohistochemistry protocols, and scoring criteria might have contributed to the slightly variable results between these studies. Given the paramount impact of IHC protocols on the positivity rates in TMA studies [18] we would not view our data as strong evidence in favor of possible ethnical differences in SENP1 expression in prostate cancers.

Our analysis revealed weak cytoplasmic SENP1 staining in secretory cells of normal prostate epithelium, while more intense cytoplasmic and nuclear staining was rare and occurred in only about 10 % of normal tissues. Finding a markedly higher fraction of cytoplasmic/nuclear SENP1 staining in cancer as compared to normal prostate suggests that SENP1 becomes upregulated in a fraction of tumors. Comparable to our observation, Li et al. [16] reported a gradual increase of SENP1 positivity from normal prostate (4.2 %) to prostatic intraepithelial neoplasia (PIN, 57.9 %) and cancer (76.5 %). SENP1 expression was significantly linked to adverse tumor features including advanced stage, high Gleason grade, and presence of lymph node metastases, preoperative PSA levels, and early biochemical recurrence in our analysis. These findings are in line with earlier studies in prostate cancer reporting significant associations with advanced and high-grade cancers as well as poor prognosis in Asian patients [16, 17]. Similar results have also been observed in analyses of other solid cancer types, including cancers of the colon [11], bladder [12], head & neck [13], and lung [14], where SENP1 overexpression was consistently linked to advanced and high-grade cancers and in some studies also with adverse clinical outcome [11, 13]. A relevant tumor biological role of SENP1 is also supported by our observation that SENP1 expression was linked to increased cell proliferation. Known biological functions of SENP1 are consistent with a role in cancer development and progression. SENP1 activity affects the homeostasis of post-transcriptional SUMO modification of various target proteins required for normal cell physiology. While both loss of SUMO conjugation as well as excessive SUMOylation results in embryonic lethality [28, 29], more subtle changes of the SUMOylation machinery lead to deregulation of multiple cellular pathways including those with relevance for cell proliferation and differentiation [10]. Genes and pathways known to be targeted by SENP1 include histone deacetylases [7], c-Jun- and ERK-dependent transcription [30, 31], cyclin D1 activity [32], Pi3K/AKT signaling pathway [33, 34], and HIF1α-dependent angiogenesis [29].

The high number of tumors in our TMA enabled us to profoundly evaluate SENP1 in the context of key genomic alterations of prostate cancer. Gene fusions involving the androgen-regulated serine protease TMPRSS2 and ERG, a member of the ETS family of transcription factors, occur in about 50 % of prostate cancers and result in strong AR-driven ERG protein overexpression [35, 36] and massive transcriptional changes [37–40]. The increased SENP1 expression levels in ERG positive cancers detected by two independent approaches (i.e. ERG-IHC and -FISH) in our study apparently reflects the AR dependency of both SENP1 and ERG, since SENP1 functions both as a transcriptional target as well as an inducer of AR expression in a positive feedback loop [32, 41].

Further subgroup analyses targeted highly recurrent chromosomal deletions that are tightly linked to the ERG status and that may delineate important molecular subgroups within ERG positive and ERG negative cancers. For example, 3p13 and *PTEN* deletions are linked to ERG positivity and deletions at 5q21 and 6q15 to ERG negativity and all these deletions have high prognostic impact within these subgroups [23–25, 42–44]. This analysis revealed that SENP1 expression was not only linked to a positive ERG status but to an even stronger extent to *PTEN* deletions. The classical function of PTEN involves control of the PI3K/AKT signaling pathway by antagonizing PI3K activity [45]. A functional relationship of PTEN and SENP1 is conceivable because SENP1 induced SUMOylation is known to occur and to have biological impact in the PTEN/PI3K/AKT signaling pathway [33, 34]. Comparison of large enough molecularly defined subgroups with clinical data is one approach to further interrogate functional interrelationships “in vivo”. The complete lack of a difference in clinical outcome between *PTEN* deleted cancers with and without SENP1 expression argues against a clinically relevant cooperative effect of reduced PTEN function and SENP1 activation. The very strong association between SENP1 overexpression and *PTEN* would, however, be consistent with models suggesting a role of SENP1 activation for development of *PTEN* deletions. This could be driven by the effect of SENP1 on histone modification and its impact on the epigenetic machinery. Both histone configuration and epigenetic events are thought to predispose to the development of specific genomic alterations including deletions [46–48]. In such a scenario, the additional *PTEN* deletion would result in such a strong disruption of cancer cell physiology that SENP1 expression no longer has a critical additional effect on tumor aggressiveness.

The overall prognostic impact of SENP1 expression was – although statistically highly significant - rather small in absolute numbers. Several models for multivariate analyses were used in this study in order to - as much as possible - model the application of prognostic features in pre- and postoperative scenarios. Unfortunately, in the real world, prognostic molecular features can hardly be analyzed on preoperative biopsies because these are typically distributed among many different pathology laboratories, and even if they were available for analyses such precious collection of tissues would be used up after only a few studies. The application of multivariate models revealed that SENP1 largely lacked independent prognostic value if all tumors and the classical molecular subgroups of ERG positive and ERG negative cancers were analyzed. However, our subgroup analyses demonstrated that the significant impact of SENP1 expression on outcome was entirely driven by the subgroup of ERG positive *PTEN* non-deleted cancers. Accordingly, independent prognostic relevance was seen for SENP1 expression in this particular subgroup. In earlier studies, we have identified other molecular markers that seemed to exert their prognostic impact only in specific molecularly defined subgroups such as in ERG positive and *PTEN* deleted cancers [49, 50], ERG negative cancers lacking *PTEN* deletion [51], ERG positive cancers [25], ERG negative cancers [52], cancers lacking *PTEN* deletion [53, 54], or in all cancers irrespective of ERG and *PTEN* status [55].

The frequent finding of subtype specific prognostic features challenges the concept of molecular classifiers that apply to all prostate cancers. For example, several multiparametric prognostic tests were recently suggested in prostate cancer [56–59] and several tests are now commercially available to patients [60, 61]. It might be interesting to see, how these tests perform in molecularly defined prostate cancer subgroups.

With SENP1 being one of the most important de-SUMOylating enzymes, it has been hypothesized that targeting SENP1 with inhibitory drugs may restore the balance of the SUMO modification system [10], and several experimental SENP1 specific inhibitors have been successfully designed as to yet [8, 62–64]. Such inhibitors may even cooperate with other treatment modalities that are commonly used in prostate cancer. Recently, Wang et al. used RNAi for depletion of SENP1 in lung cancer cell lines and found that inhibition of SENP1 markedly enhanced the radiosensitivity of lung carcinoma by promoting irradiation-induced cell cycle arrest, γ-H2AX expression and apoptosis [14]. Although clinical studies are so far lacking, these first attempts emphasize the potential druggability of SENP1 in human cancers. Given that prostate cancer is characterized by AR-driven SENP1 expression, it is possible that drugs targeting SENP1, possibly in combination with anti-androgenic therapy, will also be effective in prostate cancer.

## Conclusions

Overall, our study demonstrates that SENP1 overexpression is frequent in ERG positive prostate cancer and linked to *PTEN* deletions. Moreover, SENP1 overexpression has strong prognostic value in the subset of ERG-positive prostate cancers lacking *PTEN* deletions.

## Additional files

Additional file 1: Table S1. Association between SENP1 immunostaining results and prostate cancer phenotype in *ERG*–fusion negative tumors. (DOC 63 kb)
Additional file 2: Table S2. Association between SENP1 immunostaining results and prostate cancer phenotype in *ERG*–fusion positive tumors. (DOC 63 kb)

## Abbreviations

SUMO: Small ubiquitin-like modifiers
SENP1: SUMO1/Sentrin specific peptidase 1
ERG: v-ets avian erythroblastosis virus E26 oncogene related
PSA: Prostate-specific antigen
FISH: Fluorescence In Situ Hybridization
PTEN: Phosphatase and tensin homolog
TMA: Tissue micro array
CHD1: Chromodomain helicase DNA binding protein 1
MAP3K7: Mitogen-activated protein kinase kinase kinase 7
FOXP1: Forkhead box P1
Ki67: Marker of proliferation Ki-67
IHC: Immunohistochemistry
PI3K: Phosphatidylinositol-4,5-bisphosphate 3-kinase
AKT: v-akt murine thymoma viral oncogene homolog 1
HIF1α: Hypoxia-inducible factor 1-alpha
ETS family: (erythroblast transformation- specific) family of transcription factors
AR: Androgen receptor
RNAi: RNA interference
