# Supplementary material for: The prognostic value of SUMO1/Sentrin specific peptidase 1 (SENP1) in prostate cancer is limited to ERG-fusion positive tumors lacking PTEN deletion
Source: BMC Cancer. 2015 Jul 23;15:538. doi: 10.1186/s12885-015-1555-8 (PMC4512145; doi:10.1186/s12885-015-1555-8)
Supplement: Additional file 1: Table S1. — Association between SENP1 immunostaining results and prostate cancer phenotype in ERG–fusion negative tumors. (DOC 63 kb) [file 12885_2015_1555_MOESM1_ESM.doc]

**Additional file 1: Table S1:** Association between SENP1 immunostaining results and prostate cancer phenotype in *ERG*–fusion negative tumors

| **Parameter** |  | **SENP1** | | | | **p value** |
| --- | --- | --- | --- | --- | --- | --- |
|  | **n evaluable** | **negative (%)** | **weak (%)** | **moderate (%)** | **strong (%)** |  |
| **All cancers** | 4,742 | 71.4 | 11.4 | 11.3 | 5.9 |  |
|  |  |  |  |  |  |  |
| **Tumor stage** |  |  |  |  |  | *<0.0001* |
| pT2 | 3,186 | 73.8 | 10.3 | 10.6 | 5.3 |  |
| pT3a | 958 | 69.0 | 13.3 | 11.3 | 6.5 |  |
| pT3b-4 | 584 | 62.2 | 14.4 | 15.2 | 8.2 |  |
|  |  |  |  |  |  |  |
|  |  |  |  |  |  |  |
| **Gleason grade** |  |  |  |  |  | *<0.0001* |
| ≤3+3 | 994 | 79.9 | 7.6 | 7.4 | 5.0 |  |
| 3+4 | 2,704 | 71.2 | 11.0 | 11.6 | 6.2 |  |
| 4+3 | 760 | 64.1 | 15.4 | 14.3 | 6.2 |  |
| ≥4+4 | 265 | 62.6 | 17.7 | 14.0 | 5.7 |  |
|  |  |  |  |  |  |  |
| **Lymph node metastasis** |  |  |  |  |  | *0.2801* |
| N0 | 2,782 | 69.1 | 11.9 | 12.6 | 6.4 |  |
| N+ | 256 | 64.1 | 14.1 | 16.0 | 5.9 |  |
|  |  |  |  |  |  |  |
| **Preop. PSA level (ng/ml)** |  |  |  |  |  | *0.0414* |
| <4 | 491 | 67.2 | 12.8 | 12.6 | 7.3 |  |
| 4-10 | 2,807 | 73.1 | 10.4 | 10.8 | 5.7 |  |
| 10-20 | 1,023 | 69.0 | 13.4 | 12.5 | 5.1 |  |
| >20 | 380 | 70.0 | 11.1 | 11.3 | 7.6 |  |
|  |  |  |  |  |  |  |
| **Surgical margin** |  |  |  |  |  | *0.4836* |
| negative | 3,764 | 71.4 | 11.5 | 11.4 | 5.6 |  |
| positive | 897 | 70.6 | 11.1 | 11.3 | 7.0 |  |
|  |  |  |  |  |  |  |
