# Supplementary material for: The prognostic value of SUMO1/Sentrin specific peptidase 1 (SENP1) in prostate cancer is limited to ERG-fusion positive tumors lacking PTEN deletion
Source: BMC Cancer. 2015 Jul 23;15:538. doi: 10.1186/s12885-015-1555-8 (PMC4512145; doi:10.1186/s12885-015-1555-8)
Supplement: Additional file 2: Table S2. — Association between SENP1 immunostaining results and prostate cancer phenotype in ERG–fusion positive tumors. (DOC 63 kb) [file 12885_2015_1555_MOESM2_ESM.doc]

**Additional file 2: Table S2:** Association between SENP1 immunostaining results and prostate cancer phenotype in *ERG*–fusion positive tumors

| **Parameter** |  | **SENP1** | | | | **p value** |
| --- | --- | --- | --- | --- | --- | --- |
|  | **n evaluable** | **negative (%)** | **weak (%)** | **moderate (%)** | **strong (%)** |  |
| **All cancers** | 3,717 | 58.2 | 13.3 | 19.3 | 9.1 |  |
|  |  |  |  |  |  |  |
| **Tumor stage** |  |  |  |  |  | *0.0032* |
| pT2 | 2,209 | 60.8 | 12.2 | 18.8 | 8.3 |  |
| pT3a | 993 | 54.6 | 14.0 | 20.4 | 11.0 |  |
| pT3b-4 | 501 | 54.3 | 17.0 | 20.0 | 8.8 |  |
|  |  |  |  |  |  |  |
|  |  |  |  |  |  |  |
| **Gleason grade** |  |  |  |  |  | *<0.0001* |
| ≤3+3 | 787 | 64.3 | 10.8 | 15.9 | 9.0 |  |
| 3+4 | 2,215 | 58.3 | 12.6 | 20.1 | 9.0 |  |
| 4+3 | 563 | 52.0 | 16.3 | 21.5 | 10.1 |  |
| ≥4+4 | 134 | 47.8 | 27.6 | 18.7 | 6.0 |  |
|  |  |  |  |  |  |  |
| **Lymph node metastasis** |  |  |  |  |  | *0.0107* |
| N0 | 2,156 | 54.7 | 13.3 | 21.1 | 10.9 |  |
| N+ | 225 | 48.9 | 21.8 | 20.4 | 8.9 |  |
|  |  |  |  |  |  |  |
| **Preop. PSA level (ng/ml)** |  |  |  |  |  | *0.0028* |
| <4 | 510 | 61.6 | 11.2 | 18.6 | 8.6 |  |
| 4-10 | 2,262 | 58.8 | 12.4 | 20.4 | 8.4 |  |
| 10-20 | 674 | 55.3 | 16.2 | 17.5 | 11.0 |  |
| >20 | 230 | 51.7 | 20.0 | 17.4 | 10.9 |  |
|  |  |  |  |  |  |  |
| **Surgical margin** |  |  |  |  |  | *0.2712* |
| negative | 2,913 | 59.0 | 12.8 | 19.3 | 9.0 |  |
| positive | 736 | 55.8 | 15.4 | 19.6 | 9.2 |  |
|  |  |  |  |  |  |  |
